# Disordered eating attitude and associated factors among high school adolescents aged 12–19 years in Addis Ababa, Ethiopia: a cross-sectional study

**DOI:** 10.1186/s13104-016-2318-6

**Published:** 2016-12-07

**Authors:** Belachew Yirga, Yalemzewod Assefa Gelaw, Terefe Derso, Molla Mesele Wassie

**Affiliations:** 1 Department of Human Nutrition, College of Medicine and Health Sciences, Institute of Public Health, University of Gondar, Gondar, Ethiopia; 2 Department of Epidemiology and Biostatics, College of Medicine and Health Sciences, Institute of Public Health, University of Gondar, Gondar, Ethiopia

**Keywords:** Disordered eating attitude, Eating attitude test, Adolescent, High school

## Abstract

**Background:**

Eating disorders are very complex, frequently developed and have a public health impact on adolescents. Different studies revealed that eating disorders is a pressing public health problem among adolescents. Eating disorders may also lead to mortality due to their physiological sequelae. There is no previous study regarding disordered of eating attitude in Ethiopian adolescents. Therefore, this study aimed to assess prevalence of disordered eating attitude and its associated factors among adolescents in Addis Ababa high schools.

**Methods:**

A school-based cross sectional study was conducted. Data were collected among 836 high school adolescents aged 12–19 years from May to June, 2015 in Addis Ababa city. The data were collected by self-administered questionnaire containing eating attitudes test-26 items (EAT-26) and socio-demographic factors. Binary logistic regression analysis was carried out to identify factors associated with disordered eating attitude. Both crude odds ratio and adjusted odds ratio were calculated to show the strength of association. In multivariable analysis, variables with a *P* value of <0.05 were considered statistically significant.

**Results:**

The prevalence of disordered eating attitude among adolescents was 8.6% [95% CI 4.9, 12.3]. Being female [AOR = 1.75, 95% CI 1.03, 3.00], Mother’s educational status (Primary [AOR = 0.28, 95% CI 0.11, 0.78], Certificate/diploma [AOR = 0.22, 95% CI 0.07, 0.58] and first degree and above [AOR = 0.16, 95% CI 0.07, 0.40]) were found to be significantly associated with disordered eating attitude.

**Conclusion:**

The finding of this study revealed that a significant number of adolescents were susceptible to developing disordered eating attitude. Being female and Mothers’ education status were significantly associated with disordered eating attitude among adolescents. Provision of screening test for eating disorders focusing on female adolescents is highly recommended.

## Background

Adolescence is an intense anabolic period when requirements for all nutrients increase noticeably [1, 2]. Adolescents frequently develop eating disorders and have major public health impact [3]. Eating disorders refer to a variety of psychological illnesses associated with significant disturbances in eating attitudes and behaviors, such as eating extremely small or large amounts of food [4]. Adolescents with eating disorder have high risk to develop consequences, such as anxiety disorder, cardiovascular symptoms, chronic fatigue and pain, depressive disorder, limitation in activity related to poor health, infectious diseases, insomnia, neurological symptoms, and suicidal attempts in their early adulthood [5]. Moreover, 20% of people with an eating disorder diagnosis die from their physiological sequelae [6]. Accordingly, early diagnosis and primary prevention of eating disorders, by improving self-esteem, body image and empowerment of eating attitudes can be an important solution [7].

Nonetheless, according to different studies the prevalence of disordered eating attitude has been widely documented: in Northern Israel 5% of boys and 20.8% of girls [8], Northwest Iran 24.2% [9], Tabriz City 16.7% [10], Singapore 10.5% [11], Brazil 15.76% [12], Indian 26.67% [13], Jeddah 32.9% [14] and South Africa 21.2% [15] of studied adolescents had disordered eating attitude.

Also various factors associated with disordered eating attitude have been identified in various settings. Based on literature review, the following were some of the most prominent factors that associated with disordered eating attitude: Female gender has been associated strongly with the occurrence of disordered eating attitude [16]. Peer influences, especially girls may learn attitudes and behaviors from their peers, such as the importance of being thin and dieting behaviors, through modeling, teasing, and conversations about body image and eating [18]. Besides, adolescents frequently reading magazines (Reading at least weekly) and listening to radio programs (listening more than 1 h per day) were risk for disordered eating attitude [18, 19]. High body mass index or excess weight versus normal weight was associated with an increased risk of developing disordered eating attitude [13, 14, 20]. Concerning to parents’ socio-demographic factors; fathers’ occupation (unemployed father) [8], Marital status of parents (not married) [17] and low mothers education level [11] were some important factors increasing risk of disordered eating attitude. Comments and cultural ideas regarding appearance, such as thin or muscular ideal were additional factors in the risk of disordered eating attitude [18].

There is no any study who addresses the level of disordered eating attitude in Ethiopia. Besides, there is no strategies and programs concerning disordered eating attitude in Ethiopian adolescents. This study will be the first of its kind in addressing the prevalence and associated factors of disordered eating attitude among adolescents of Addis Ababa high schools.

## Methods

### Study setting and design

Cross sectional study was conducted from May to June, 2015 in Addis Ababa high schools. Addis Ababa is the capital city of Ethiopia with an area of 530 km^2^ and a total population of 3 million. Also Addis Ababa city administration is divided into ten administrative sub-cities.

### Study participants and sampling procedure

All high school adolescents aged 12–19 years who attended high schools in Addis Ababa were included in the study. To estimate disordered eating attitude among adolescents aged 12–19 years, sample size was calculated using Epi-info version 3.7 by considering the following assumptions; 50% prevalence of expected disordered eating attitude since there was no previous study conducted in the country, 95% level of confidence, 5% margin of error, 10% non-response rate and a design effect of 2. Thus, a minimum sample size of 847 was obtained.

Regarding to sampling technique, firstly two sub cities (Arada and Gullele) were selected randomly using lottery method among ten Sub cities of Addis Ababa. Then, two schools, Lideta catholic cathedral high school with a total number of 1679 students from Arada and Dileber high school with a total number of 1267 students from Gullele sub city were schools selected by randomly. The sampling frame of students in each of the grade level and section was obtained from the academic director offices of schools.

Lastly, students were selected from each school and grade level (9–12) using a systematic sampling technique after identifying an initial starting student by use of a random number. The sample sizes were distributed to each grade and section proportionally according to the class size of the grade level. Adolescents in the selected grade level were further selected and given self-administrated questionnaires.

### Data collection tools and procedure

Data were collected using structured, pretested, and self- administered questionnaire to obtain socio-demographic information and disordered eating attitude. The questionnaire was first translated from English to Amharic and retranslated back to English by language experts to maintain its consistency. Final the questionnaire was administered in Amharic for data collection. Five data collectors and two field supervisors were recruited for the study. One day exhaustive training regarding the objective of the study, confidentiality of information, and data collection techniques was given to data collectors and supervisors. The questionnaire was piloted on 5% (42) school adolescents out of the study area. During pre-test, the acceptability and applicability of the procedures and tools were evaluated. All filled questioners were checked for completeness, accuracy and consistency by the supervisor and the primary investigator.

### Operational definitions and study variables

The outcome variable of the study was disordered eating attitude. Eating attitude was determined by using eating attitude test-26 (EAT-26) items which were grouped into three factors from subscales [21]. Factor I is ‘dieting’ with 13 subscales; “example, terrified of being overweight”, Factor II is ‘bulimia and food preoccupation’ with six subscales; “example, find myself preoccupied with food” and Factor III is ‘oral control’ with seven subscales; “example, avoid eating when I am hungry. For all items except #26, each of the responses receives the following value (six-point scale from ‘always’ to ‘never’): with 3 points to ‘always’, 2 points to ‘very often’, 1 point to ‘often’, and 0 points to ‘sometimes, rarely and never’. Thus, eating attitude was computed with a maximum score of seventy-eight (78). The EAT-26 scores of 20 or higher defined as disordered eating attitude (unfavorable eating attitude) and below 20 perceived as favorable eating attitude.

#### Independent variables

The independent variables incorporated in the study were: socio demographic characteristics, environmental influences (parental influence, peer pressure and media) and nutritional status.

### Data analysis

Data was checked, coded and entered to Epi-info version 7 and was exported to SPSS (Statistical Package for Social science) version 20 for analysis. Reliability analysis was performed to ensure that items with in each factor were consistent. Internal consistency was checked by calculating Cronbach’s alpha for each of the items to examine the extent to which adolescents answered consistently to the items in each of the three factors. Descriptive and analytical statistics including bivariate and multivariate analysis was employed. All variables with a P value of less than 0.2 in bivariate analysis were entered to multivariate analysis to control the possible effect of confounders. Both crude odds ratio (COR) and adjusted odds ratio (AOR) were estimated to show the strength of association. In multivariate analysis, variables with a P value of ≤0.05 were considered as statistically significant.

## Results

### Socio-demographic characteristics of participants

A total of 836 adolescents aged 12–19 were enrolled in the study with the response rate of 98.6%. The mean age (±SD) of students was 16.48 (±1.37) with two-third (66.1%) in the age group of 16–18 years. More than half (56.6%) of the study participants were females. Regarding the ethnicity of the respondent’s majority (43.9%) was Amhara. About three-fourth 76.1 and 74.2% respondents were Orthodox Christian followers and attended their education in private school, respectively. Vast majority of (87.2%) of the adolescents were living with their family. Three hundred seventy-five (44.86%) participants were grade 9 (Table 1).

**Table 1:** Socio-demographic and economic characteristics of adolescents aged 12–19 years in Addis Ababa high schools, 2016

Variables	Frequency	Percent
Sex		
Female	473	56.6
Male	363	43.4
Age		
12–15	245	29.3
16–18	553	66.1
19	38	4.5
Mean (±SD) age	16.48 (±1.37)	
Religion		
Orthodox	636	76.1
Protestant	137	16.4
Muslim	38	4.5
Others^a^	25	3
Ethnicity		
Amhara	367	43.9
Oromo	162	19.4
Tigrawaye	145	17.3
Gurage	112	13.4
Others^b^	50	6
Grade level		
Grade 9	375	44.9
Grade 10	93	11.1
Grade 11	126	15.1
Grade 12	242	28.9
Students Living with		
Mothers and fathers	729	82.2
Others^c^	107	12.8
Mother educational status		
No schooling	31	3.7
Primary	73	8.7
Secondary school	71	8.5
Certificate/diploma	204	24.4
Degree (first or above)	457	54.7
Father education status		
No schooling	16	1.9
Primary school	55	6.6
Secondary school	41	4.9
Certificate/diploma	103	12.2
Degree (first or above)	622	74.4
Mother’s occupation		
Governmental and private employed	344	41.1
House wife	102	12.2
Merchant	220	26.3
Others^d^	170	20.3
Father’s occupation		
Governmental and private employed	334	40
Merchant	262	31.3
Others^d^	240	28.7
School type		
Government	620	74.2
Private	216	25.8
BMI kg/m^2^		
<18.5	134	16
18.5–25	601	71.9
>25	101	12.1

^a^Catholic, Gehova

^b^Walayta, Hadya, Sidama

^c^Sister, brother, guardian, grand mothers or fathers

^d^Daily laborer, private work

### Prevalence of disordered eating attitude

The Mean (±SD) score of EAT-26 was 9.1 ± 7.49. Besides, the Mean (±SD) score of EAT-26 subgroups, i.e., Dieting, Bulimia and Food Preoccupation, and Oral Control were 5.31 ± 0.82, 1.30 ± 0.63 and 2.54 ± 0.81, respectively. The results of the study revealed that 8.6% [95% CI 4.9, 12.3] of the students had disordered eating attitude. The prevalence of disordered eating attitude was higher in female adolescents 50 (5.98%) (P = 0.04) as compared with male adolescents 22 (2.62%). About 8.7 and 4.2% of adolescents was for “I Am terrified about being overweight” always and very often, respectively. Only 1.3 and 1.2% of adolescents was for “Have the urge to vomit after meals” always and very often, respectively. Besides, 7.3 and 4.3% of adolescents was for “I am preoccupied with the thought with of having fat on my body” always and very often, respectively (Table 2).

**Table 2:** EAT-26 score among adolescents aged 12–19 years in Addis Ababa high schools, 2016

Factor I (dieting)	Always N, (%)	Very often N, (%)	Often N, (%)	Sometimes, rarely and never N, (%)
Terrified of being overweight	73 (8.7)	35 (4.2)	43 (5.1)	685 (81.9)
Aware of the calorie content of foods that I eat	129 (15.4)	85 (10.2)	85 (10.2)	537 (64.2)
Particularly avoid food with high carbohydrate content	50 (6)	48 (5.7)	59 (7.1)	679 (81.2)
Feel extremely guilty after eating	15 (1.8)	11 (1.5)	25 (3)	785 (93,9)
I am extremely preoccupied with a desire of thinner	77 (9.2)	25 (3)	49 (5.9)	685 (81.9)
Think burning up calories when I exercise	60 (7.1)	23 (2.8)	81 (9.7)	672 (80.4)
Other people think that I am too thin	38 (4.5)	25 (3)	29 (3.5)	744 (89)
Avoid food with sugar in them	29 (3.5)	51 (6.1)	85 (10.2)	671 (80.3)
Eat diet food	40 (4.8)	29 (3.5)	42 (5)	725 (86.7)
Feel uncomfortable after eating sweets	35 (4.2)	26 (3.1)	58 (6.9)	717 (85.8)
Engage in dieting behavior	38 (4.5)	24 (2.9)	48 (5.7)	72 (86.8)
Like my stomach to be empty	32 (3.8)	39 (4.7)	52 (6.2)	713 (85.3)
Enjoy trying new rich food	162 (19.4)^a^	199 (23.8)^b^	201 (24)^c^	274 (32.8)^d^
Factor II (Bulimia and food preoccupied)				
Find myself preoccupied with food	44 (5.3)	57 (6.8)	44 (5.3)	691 (82.7)
Have gone on eating binges where I feel that I may not be able to stop	38 (4.5)	58 (6.9)	43 (5.2)	697 (83.4)
Vomit after I have eaten	5 (0.6)	9 (1.1)	12 (1.4)	810 (96.9)
Feel that food control my life	33 (3.9)	28 (3.3)	65 (7.8)	710 (84.9)
Give too much time and thought to food	27 (3.2)	30 (3.6)	39 (4.7)	740 (88.5)
Have the urge to vomit after meals	11 (1.3)	10 (1.2)	4 (0.5)	811 (97)
Factor III (oral control)				
Avoid eating when I am hungry	20 (2.4)	32 (3.8)	37 (4.4)	747 (89.4)
Cut my food into pieces	57 (6.6)	128 (15.3)	68 (8.1)	583 (69.7)
Feel that others would prefer if I ate more	63 (7.5)	51 (6.1)	80 (9.6)	642 (76.8)
I am preoccupied with the thought with of having fat on my body	61 (7.3)	36 (4.3)	66 (7.9)	673 (80.5)
Take longer than other to eat my meal	25 (3)	41 (4.9)	82 (9.8)	688 (82.3)
Display self-control around food	27 (3.2)	23 (2.8)	54 (6.5)	732 (87.6)
Feel that others pressure me to eat	58 (6.9)	42 (5)	86 (10.3)	658 (77.8)

^a^Never

^b^Rarely

^c^Sometimes

^d^Always, very often, often

### Factors associated with disordered eating attitude

In bivariate analysis sex, age, mothers education, mothers occupation, fathers occupation, school type and body mass index were found with a p value of less than 0.2.

However, the result of multivariate analysis showed that student’s sex and mother’s education were independently and significantly associated with disordered eating attitude. There was a significant negative association between mother’s education and the adolescents’ disordered eating attitude. The odds of developing disordered eating attitude was lower in adolescents from parents with primary and secondary school [AOR = 0.28, 95% CI 0.11, 0.78], certificate/diploma [AOR = 0.22, 95% CI 0.07, 0.58] and degree [AOR = 0.16, 95% CI 0.07, 0.40] was lower as compared with adolescents from parents with no schooling. However, the odds of eating disorder was 1.75 times higher among female adolescents as compared to males [AOR = 1.75, 95% CI 1.03, 3.00] (Table 3).

**Table 3:** Factors associated with disordered eating attitude among adolescents aged 12–19 years in Addis Ababa high schools, 2016

Variables	Disordered eating attitude		Crude odds ratio (95% CI)	Adjusted odds ratio (95% CI)	P value
	Yes	No			
Sex					
Female	50	423	1.83 (1.09, 3.09)	*1.75* (*1.03, 3.00*)	*0.040*
Male	22	341	1	1	
Age					
≤15	28	217	1.60 (0.97, 2.64)	1.03 (0.55, 1.95)	
>15	44	547	1	1	
Mother education					
No schooling	9	22	1	1	
Primary and secondary school	14	130	0.26 (0.10, 0.68)	*0.28* (*0.11, 0.78*)	*0.014*
Certificate/diploma	17	187	0.22 (0.09, 0.56)	*0.22* (*0.07, 0.58*)	*0.002*
Degree	32	425	0.18 (0.08, 0.43)	*0.16* (*0.07, 0.40*)	*0.000*
Mothers occupation					
Employed	39	305	2.05 (0.99, 4.21)	1.83 (0.86, 3.87)	
House wife	8	94	1.36 (0.52, 3.57)	0.84 (0.30, 2.36)	
Merchant	15	205	1.17 (0.51, 2.68)	1.10 (0.47, 2.58)	
Others	10	160	1	1	
Father occupation					
Employed	36	298	1.69 (0.92, 3.13)	1.4 (0.47, 2.76)	
Merchant	20	242	1.16 (0.56, 2.29)	1.46 (0.51, 4.19)	
Others	16	224	1	1	
School type					
Private	46	574	1.71 (1.03, 2.84)	1.76 (0.80, 3.83)	
Government	26	190	1	1	
BMI					
<18.5	19	115	1	1	
18.5–25	47	554	0.51 (0.29, 0.91)	0.58 (0.32, 1.06)	
>25	6	95	0.32 (0.15, 0.99)	0.41 (0.15, 1.09)	

Italic values indicate significant P value(0.05)

## Discussion

The results of the current study demonstrated that 8.6% [95% CI 4.9, 12.3] of the students had unfavorable eating attitudes. This finding was harmonious with a study conducted in Singapore 10.5% [11]. However, the finding was lower than the results of previous studies; in Northern Israel 5% of boys and 20.8% of girls [8], Northwest Iran 24.2% [9], Tabriz City 16.7% [10], Brazil 15.76% [12], Indian 26.67% [13] and South Africa 21.2% [15]. This observed discrepancy could be due to socio-demographic and cultural variations in the study areas. Moreover, measures used to assess disordered eating attitudes may also be the possible explanations for the observed variations. The high prevalence of overweight and obesity may be another important reason for high prevalence of eating attitude disorders in Israel, Brazil and South Africa. Difference in media exposure and feeding habit could also be the possible explanation for the observed differences [18, 19].

In the current study, those adolescents from educated parents were less likely to develop unfavorable eating attitude. This result was similar with the study finding in Singapore, which revealed that the students with disordered eating attitudes were less likely to have parents with higher education [11]. This might be due to the fact that those from educated family will have right information about the consequences of eating disorders and immune from developing favorable attitude towards eating attitude disorders. However, this finding is not consistent with a study done in China were adolescents with educated parents showed disordered eating attitudes [22]. This might be due to the fact that double burden of malnutrition in becoming common in china and those from well doing family had high chance of developing obesity which further contributes to the development of disordered attitude towards diet in adolescents. The current study revealed that female Adolescents were 1.75 times more likely to have eating attitude disorders compared to their male counterparts. This result supported with other findings [16, 22]. Female are more concerned about their body image which may lead to development of eating attitude disorders. The Cultural pressures that glorify thinness and narrow definitions of beauty that include only girls of specific body weights and shapes has significant implication on development of eating disorders in girls. Media is the most suitable source for health related information for adolescent girls. However, the messages from this media my promote thinness as good indicator of girls beauty and acceptance.

## Conclusion

As no prior study in the country, the prevalence of our study publicized to be more adolescents was susceptible to eating disorders. Mothers’ educational status and sex of adolescents were found to be important predictor of developing eating attitude disorders among high school adolescents. Provision of early screening and timely treatment of female adolescents with eating attitude disorders is highly recommended. Further research is needed to develop intervention programs to control eating disorders among Ethiopian adolescents.

## Abbreviations

AOR: adjusted odds ratio
EAT-26: eating attitude test-26
CI: confidence interval
COR: crude odds ratio
SD: standard deviation
SPSS: statistical package for social science
